# The accuracy of fatty liver index for the screening of overweight and obese children for non-alcoholic fatty liver disease in resource limited settings

**DOI:** 10.1186/s12887-022-03575-w

**Published:** 2022-08-30

**Authors:** Mawanane Hewa Aruna Devapriya de Silva, Ruwani Punyakanthi Hewawasam, Chanika Roshini Kulatunge, Rathnayake Mudiyanselage Anjali Chamika

**Affiliations:** 1 Department of Paediatrics, University of Ruhuna, Galle, Sri Lanka; 2 Department of Biochemistry, Faculty of Medicine, University of Ruhuna, Karapitiya, Galle, Sri Lanka; 3 Teaching Hospital, Karapitiya, Galle, Sri Lanka; 4 Department of Nursing, Faculty of Allied Health Sciences, University of Peradeniya, Peradeniya, Sri Lanka

**Keywords:** Childhood obesity, NAFLD, Screening, Fatty liver index, Ultrasonography, Resource limited settings

## Abstract

**Background:**

Non-alcoholic fatty liver disease (NAFLD) has become the most common liver disease among children with a higher prevalence among obese. Fatty liver index (FLI), an algorithm derived to screen NAFLD using the waist circumference, body mass index, triglyceride and gamma glutamyl-transferase concentration was rarely investigated in Asian paediatric population. Since each component is routinely measured in clinical practice, it is feasible to measure FLI even in resource limited settings. Hence, we determined the accuracy of FLI for the screening of NAFLD in obese children in Southern Sri Lanka.

**Methods:**

A descriptive cross sectional study was conducted on ninety five children (56 boys) aged 5–15 years with BMI ≥ 85th percentile for age and gender based on CDC 2000 growth charts recruited from the nutrition clinic at the Teaching Hospital, Karapitiya, Sri Lanka. NAFLD was diagnosed by ultrasonography. Factors associated with ultrasonographic fatty liver such as biochemical parameters and fatty liver index in either sex or the whole population were determined by a multivariate analysis. The ability of FLI to screen NAFLD was determined by the analysis of area under the receiver operator characteristic curve (AUROC) and the maximum Youden index analysis.

**Results:**

Overweight and obese children with ultrasonographic fatty liver had a significantly higher FLI than those without fatty liver according to the multivariate analysis performed (Odds ratio 3.524; 95% CI 1.104–11.256, *P* = 0.033). AUROC of FLI for NAFLD was 0.692 (95% CI; 0.565–0.786) and the optimal cut off value for the screening of NAFLD was 30 (Maximum Youden index 0.2782, Sensitivity, 58.33%; Specificity, 69.49%).

**Conclusion:**

FLI could accurately be used in resource limited community settings and in epidemiological studies to screen overweight and obese children for NAFLD.

## Introduction

Non-alcoholic fatty liver disease (NAFLD), an emerging public health issue in both high, low- and middle-income countries, is defined as an infiltration of fat in the liver greater than 5–10% of liver weight. According to recent reports, 1 billion people are estimated to be affected by NAFLD worldwide [1]. However, limited data are available on the incidence of NAFLD in children. A systematic review and meta-analysis published in 2015 revealed the prevalence of NAFLD as 7.6% among the general paediatric population worldwide compared to 34% reported in the paediatric obese population. Diagnosis of NAFLD was based on non-invasive surrogate markers of the diseases such as serum alanine aminotransferases (ALT) and ultrasonography [2]. Based on autopsy studies, the prevalence of NAFLD was between 4.2–9.6% worldwide while it increased up to 38% in the paediatric obese population [3].

NAFLD progresses as a spectrum of diseases from uncomplicated fatty liver, nonalcoholic steatohepatitis and liver cirrhosis to hepatocellular carcinoma [4]. In a majority of children, NAFLD is associated with central or generalized obesity, insulin resistance, and dyslipidemia that is characterized by high triglyceride and low high-density lipoprotein cholesterol (HDL-C) levels [5].

Changes in the lifestyle of populations across the globe contribute significantly to the development of NAFLD. The patatin-like phospholipase domain which contain protein 3 (PNPLA3) gene has also been linked to the increased triglyceride concentration in the liver, fibrosis, and inflammation. It is currently considered as the only confirmed gene heterogeneity linked to the ethnicity associated with NAFLD [6]. It has also been widely reported that South Asians have a higher tendency to have dyslipidemia compared to Western populations due to the presence of altered body fat distributions [7, 8]. Previous studies reported that PNPLA3 single nucleotide polymorphisms were responsible for the higher hepatic fat content reported in Asian Indians [9]. South Asians are potentially different from other populations due to differences in their diet, economic status & growth, and other confounding factors [4]. A limited number of data have been reported on NAFLD patients in the South Asian region compared with other ethnic groups [10].

Since NAFLD is often asymptomatic, it is frequently identified incidentally when biochemical investigations are performed on blood or abdominal imaging, is performed for other indications. Early diagnosis is of utmost importance to prevent the progression of NAFLD to irreversible complications.

Different non-invasive indices for the diagnosis of NAFLD have been assessed against hepatic histology, the currently accepted clinical reference [5]. However, to date, such surrogate markers and prediction scores developed have not been identified as clinically useful due to insufficient validation [11]. A simple and effective diagnostic and screening methods would be useful for the early detection and better management of NAFLD in paediatric patients [12]. Bedogni et al. first introduced fatty liver index (FLI), an algorithm based on body mass index (BMI), waist circumference, triglyceride, and gamma-glutamyl-transferase (GGT) for the prediction of fatty liver [13] in an Italian cohort. Each component is routinely measured in clinical practice thus it is feasible even in resource limited settings. It has been validated as a practical, reliable and economic technique and was reported to have an excellent discriminative ability to detect NAFLD in epidemiological studies, especially in adult populations [14]. However, it has never been validated in a South Asian population and very limited data are available on its validity in paediatric obese population worldwide. We hypothesize that FLI could be validated against ultrasonography and the optimum cut off values could be used in the screening of NAFLD in obese children in resource limited settings and in epidemiological studies. Therefore, the objective of this study was to determine the accuracy of FLI in the screening of NAFLD in overweight and obese children in Southern Sri Lanka.

## Methods

### Study population

A descriptive cross sectional study was conducted on ninety five children (56 boys) aged 5–15 years who had a BMI ≥ 85th percentile for age and gender based on the Centers for Disease Control and prevention (CDC) 2000 growth charts from the Southern province of Sri Lanka. Children with concomitant liver disease, dysmorphic syndromes, those who were obese due to iatrogenic causes, those who are on steatogenic medication and oral anti-diabetic drugs were excluded from the study. Parents of all study subjects provided written informed consent. The study protocol was approved by the Ethical Review Committee of the Faculty of Medicine, University of Ruhuna, Sri Lanka. Children were recruited at the nutrition clinic at the Teaching Hospital, Karapitiya, Galle, Sri Lanka and all study subjects underwent measurement of anthropometric parameters, laboratory examination and abdominal ultrasonography.

### Anthropometric and biochemical assessment

Height was measured without shoes while the heel, buttocks, back of shoulder and occiput are kept in the vertical plane and head in horizontal Frankfurt plane using a wall-mounted stadiometer (Seca, Birmingham, UK) to the nearest 0.1 cm. The weight was measured using a calibrated electronic weighing scale (Nagata, BW-110H CAP, Taiwan) in minimal light indoor clothing, to the closest 0.1 kg. BMI was calculated as weight (Kg) divided by height (m) squared. Waist circumference was also measured at the umbilical level in the standing position to the nearest 0.1 cm [15].

After an overnight fast, at least for 10 hours, venous blood samples were collected for the determination of total cholesterol, high density cholesterol, low density cholesterol, triglyceride, alanine aminotransferase and gamma glutamyl transfearase concentrations at the Teaching Hospital, Karapitiya, Galle, Sri Lanka.

### Evaluation of NAFLD

#### Ultrasonography

Abdominal ultrasonography was used for the diagnosis of NAFLD in each study participant and it was performed by a single Consultant radiologist using high resolution B-mode ultrasound system (Canon Aplio i600, Japan) with 1–8 MHz probe at the Teaching Hospital, Karapitiya, Galle, Sri Lanka. The presence or absence of NAFLD and grading of fatty infiltration were recorded. NAFLD was diagnosed in the presence of two of the three abnormal findings; increased hepatic echogenicity compared to the renal cortex or the spleen, blurring of the liver vasculature and deep attenuation of the ultrasonographic signals. Normal liver echogenicity was graded as zero and diffusely increased hepatic echogenicity with normal visualization of the diaphragm and the intrahepatic vascular borders was graded as grade I NAFLD. Grade II NAFLD was categorized as diffusely increased hepatic echogenicity obscuring periportal echogenicity when diaphragmatic echogenicity is still appreciable. Grade III NAFLD was graded as diffusely increased hepatic echogenicity obscuring periportal as well as diaphragmatic echogenicity.

#### Fatty liver index

Triglyceride and gamma glutamyl transferase concentrations in serum and BMI and waist circumference measured in each participant were applied in the predictor algorithm mentioned below for the prediction of fatty liver [13].

$$\mathrm{FLI}=\frac{\left(\mathrm e^{0.953\;\mathrm X\;\log\;\mathrm e(\mathrm{TG})\;+\;0.139\;\mathrm X\;\mathrm{BMI}\;+\;0.718\;\mathrm X\;\log\;\mathrm e(\mathrm{GGT})\;+\;0.053\;\mathrm X\;\mathrm{WC}-15.745}\right)}{\left(1+\mathrm e^{0.953\;\mathrm X\;\log\;\mathrm e(\mathrm{TG})\;+\;0.139\;\mathrm X\;\mathrm{BMI}\;+\;0.718\;\mathrm X\;\log\;\mathrm e(\mathrm{GGT})\;+\;0.053\;\mathrm X\;\mathrm{WC}-15.745}\right)\;\mathrm x\;100}$$

FLI score below 30 rule out NAFLD, 30 < FLI > 60 indicates an intermediate risk and FLI ≥ 60 indicates high risk of NAFLD.

### Statistical analysis

Student t-test and Pearson’s chi-squared analysis were performed to compare continuous and categorical variables between subjects with and without ultrasonographic fatty liver. Continuous variables were presented as mean ± standard deviation (SD) and categorical variables were presented as numbers (proportion). Variables with statistical significance (*P* < 0.05) or proximate to it (*P* < 0.1) in univariate analysis were further included in multivariate analysis using a logistic regression model with the forward stepwise selection procedure. The area under the receiver operator characteristic curve (AUROC) was used to indicate the diagnostic accuracy of the equations and predictive values of the FLI and its individual component for diagnosing NAFLD. Comparisons between the AUROC of the FLI and its individual components were compared according to the method described by Huang et al. (2015) [12]. The AUROC was expressed as plots of the test sensitivity vs. 1-specificity. The sensitivity (SN), specificity (SP), positive likelihood ratio (LR+), negative likelihood ratio (LR−), positive predictive value (PPV), negative predictive value (NPV) and Youden index were also assessed. The point with the maximum Youden index was used as the cut-off point of the FLI for detecting NAFLD. Participants were categorized into four groups by four different diagnostic methods: non-NAFLD diagnosed based on both the FLI and hepatic US scan [FLI (−) and US (−)], NAFLD diagnosed only based on the US scan [FLI (−) and US (+)], NAFLD diagnosed only based on the FLI [FLI (+) and US (−)], and NAFLD diagnosed based on both the FLI and US method [FLI (+) and US (+)]. Comparisons of metabolic characteristics between participants with [FLI (−) and US (+)] and those with [FLI (+) and US (−)] were performed using t-test for continuous variables and the chi-squared test for categorical variables. All statistical analyses were performed using SPSS 21.0 for Windows (SPSS Inc., Chicago, IL, USA). A *P* value of < 0.05 was considered to be statistically significant.

## Results

**Fig. 1:**
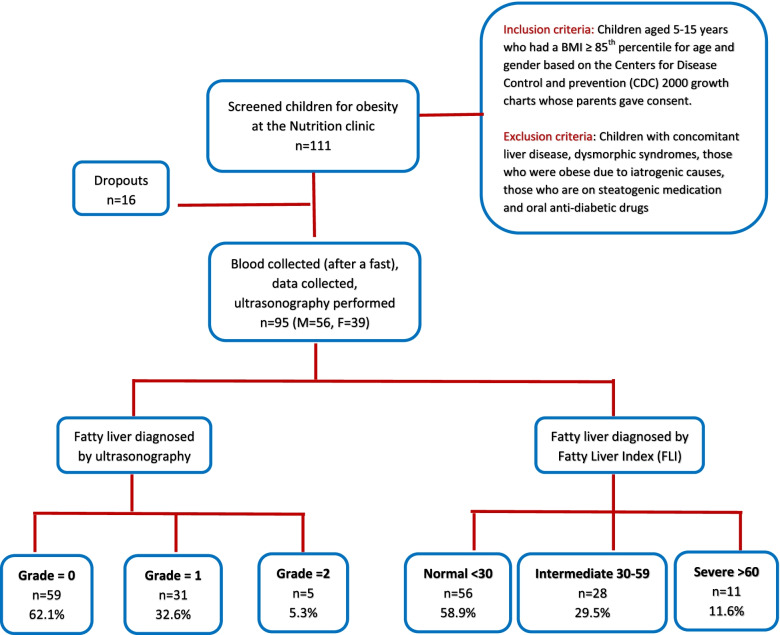
Flow chart of the study participants

Among the 95 overweight and obese children enrolled in this study, the mean age was 10.66 years and 58.9% were male. Fatty liver was diagnosed by ultrasonography in 37.9% (*n* = 36) children whereas 32.6% were diagnosed with grade 1 fatty liver and 5.3% were diagnosed with grade 2 fatty liver (Table 1). Almost similar results were obtained when NAFLD was screened with FLI where 58.9% were ruled out of having fatty liver compared to 62.1% when diagnosed with ultrasonography. However, 29.5 and 11.6% were identified as children with intermediate and severe risk of NAFLD according to the FLI calculated (Fig. 1).

**Table 1:** Distribution of non-NAFLD and NAFLD subjects diagnosed according to ultrasonography and FLI

	Frequency	Percentage
**Fatty liver grade based on ultrasonography**		
0	59	62.1
1	31	32.6
2	5	5.3
Total	95	100.0
**Fatty liver index categories**		
Normal (FLI < 30)	56	58.9
Intermediate (FLI 30–59)	28	29.5
Severe (FLI ≥60)	11	11.6
Total	95	100.0

*FLI* Fatty liver index, *NAFLD* Non-alcoholic fatty liver disease

According to Table 2, the majority of children diagnosed with NAFLD according to ultrasonography (66.7%) and identified by FLI (61.5%) were males. Body mass index, GGT concentration and ALT concentration were significantly elevated (*P* < 0.05) in the children diagnosed with NAFLD according to ultrasonography. However, body mass index, waist circumference, GGT concentration, triglyceride concentration, HDL cholesterol concentration and ALT concentration were significantly elevated (*P* < 0.05) in the children identified with NAFLD according to the FLI calculated compared to children with FLI less than 30 who are defined as normal.

**Table 2:** Basic demographic, anthropometric, biochemical characteristics of the study subjects categorized by ultrasonography and FLI

Characteristics	All (***n*** = 95)	NAFLD diagnosed by ultrasonography			NAFLD screened according to FLI		
		Without fatty liver (***n*** = 59)	With fatty liver (*n* = 36)	P	Without fatty liver (***n*** = 56)	With fatty liver (***n*** = 39)	P
Age (years),	10.66 ± 2.47	10.80 ± 2.52	10.44 ± 2.41	*p* = 0.504	10.04 ± 2.52	11.56 ± 2.12	*p* = 0.003*
**Sex, n(%)**							
Female, n(%)	39(41.1%)	27(45.8%)	12(33.3%)	*p* < 0.001*	24(42.9%)	15(38.5%)	*p* = 0.041*
Male, n(%)	56(58.9%)	32(54.2%)	24(66.7%)	*p* = 0.131	32(57.1%)	24(61.5%)	*p* = 0.131
Body mass index (kg/m2)	25.52 ± 3.71	24.92 ± 3.65	26.51 ± 3.63	*p* = 0.043*	23.77 ± 2.55	28.04 ± 3.68	*p* < 0.001*
Waist circumference (cm)	85.68 ± 9.38	84.36 ± 9.48	87.85 ± 8.92	*p* = 0.078	80.71 ± 6.91	92.82 ± 7.72	*p* < 0.001*
GGT (IU/L)	23.23 ± 13.35	19.56 ± 9.44	29.25 ± 16.47	*p* = 0.001*	19.02 ± 8.89	29.28 ± 16.21	*p* < 0.001*
Triglyceride (mg/dL)	118.11 ± 57.05	113.27 ± 53.47	126.03 ± 62.43	*p* = 0.407	97.73 ± 48.43	147.36 ± 56.26	*p* < 0.001*
Total cholesterol (mg/dL)	191.52 ± 50.50	187.24 ± 49.60	198.53 ± 51.89	*p* = 0.293	183.66 ± 52.92	202.79 ± 45.11	*p* = 0.069
HDL-cholesterol (mg/dL)	50.14 ± 9.97	49.93 ± 9.60	50.49 ± 10.67	*p* = 0.793	48.76 ± 9.75	52.13 ± 10.06	*p* = 0.106
LDL-cholesterol (mg/dL)	117.75 ± 42.43	114.65 ± 41.32	122.83 ± 44.30	*p* = 0.365	115.35 ± 44.52	121.20 ± 39.54	*p* = 0.512
ALT (IU/L)	31.65 ± 21.55	24.63 ± 13.21	43.17 ± 27.18	*p* < 0.001*	26.34 ± 17.47	39.28 ± 24.62	*p* < 0.001*

*NAFLD* Non-alcoholic fatty liver disease, *FLI* Fatty liver index, *GGT* Gamma glutamyl transferase, *HDL* High density lipoprotein, *LDL* Low density lipoprotein, *ALT* Alanine amino transferase, * significant at *P* < 0.05

**Fig. 2:**
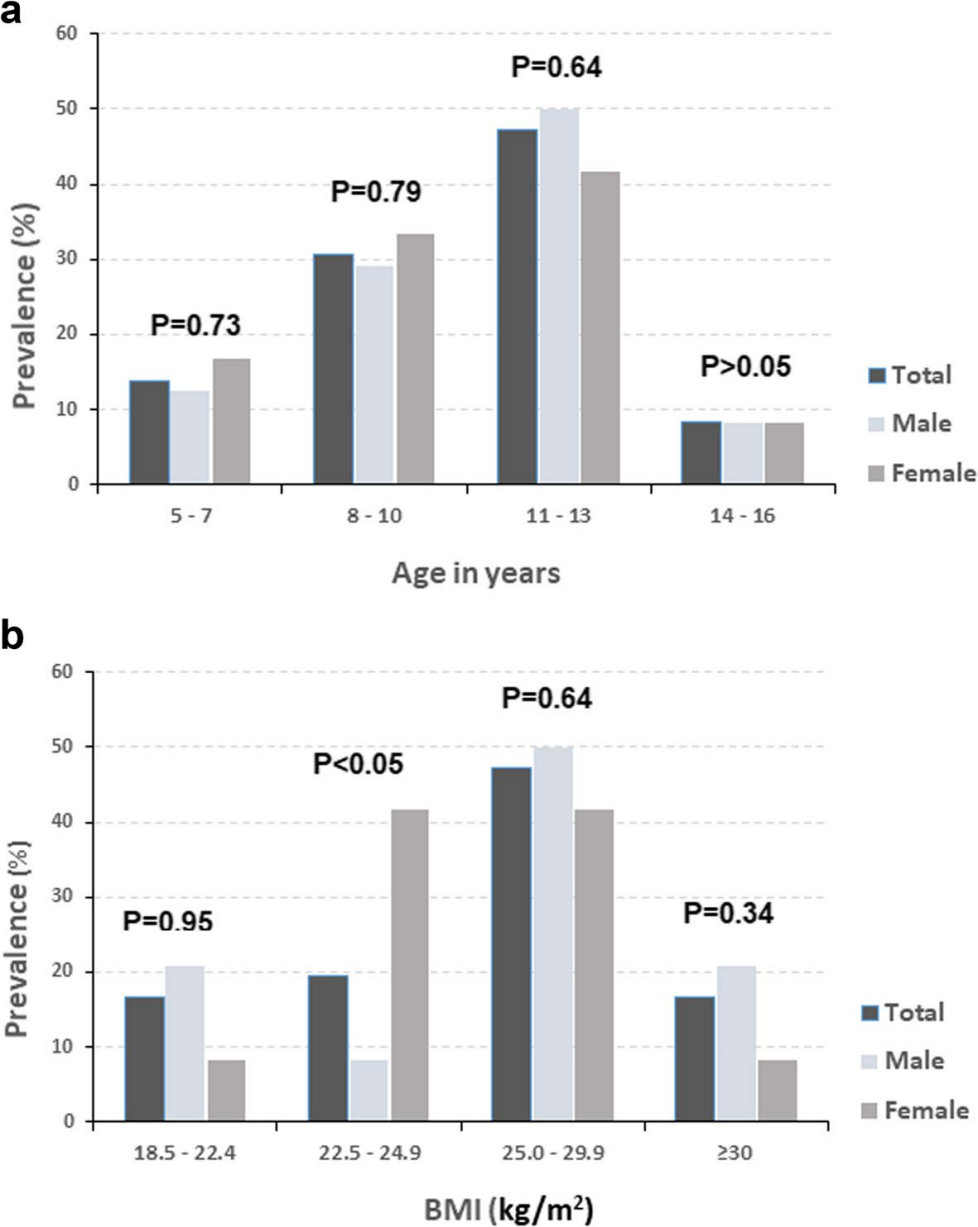
Prevalence of ultrasonographically diagnosed NAFLD in children stratified according to (**a**) age and (**b**) BMI

**Fig. 3:**
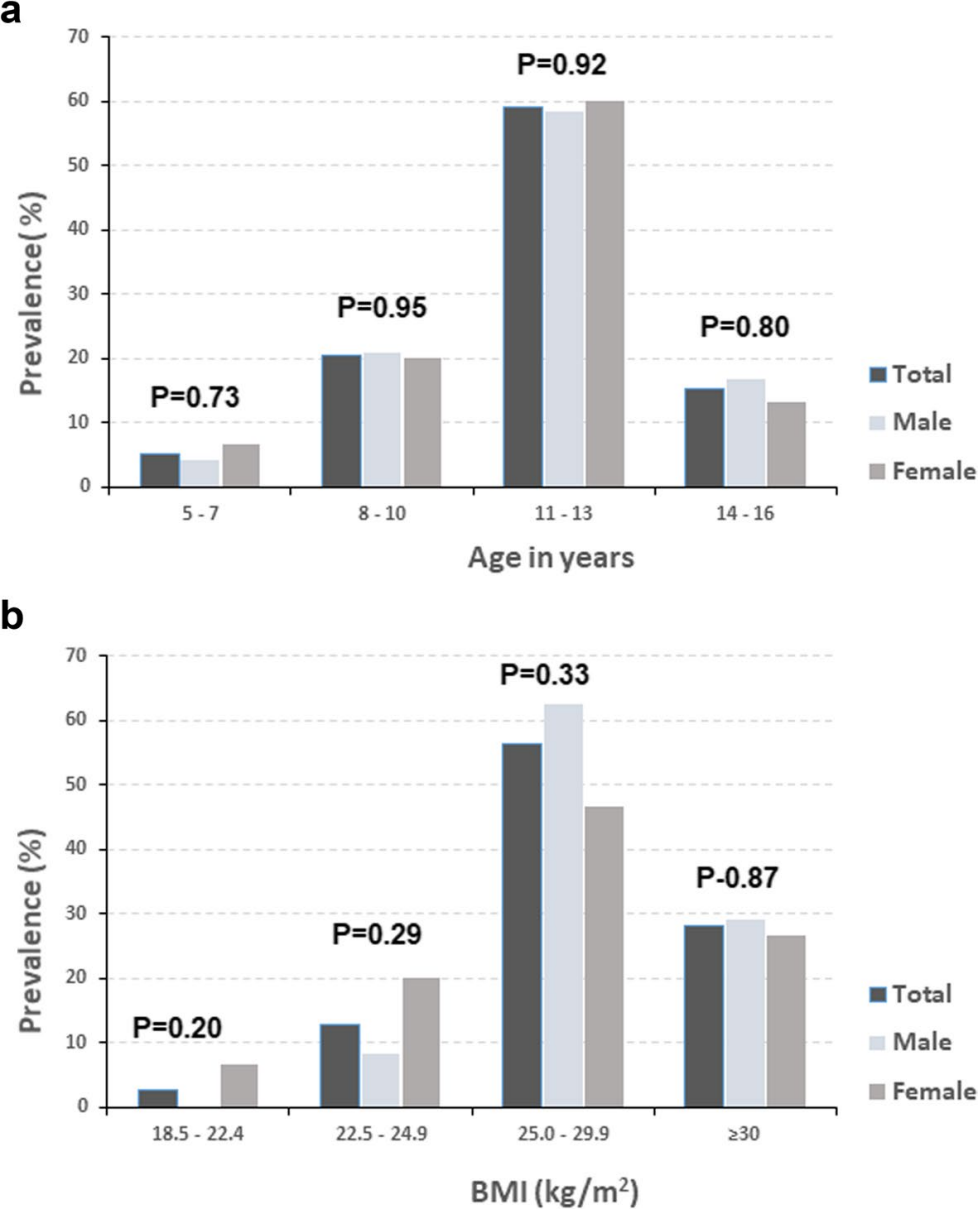
Prevalence of NAFLD screened according to FLI in children stratified according to (**a**) age and (**b**) BMI

According to ultrasonography, children in the age group 11–13 years had the highest prevalence of NAFLD and male subjects had a significantly higher rate of fatty liver diagnosed by ultrasonography (50.0% vs 41.7%, Fig. 2). When stratified by BMI, children with BMI in the range 25.0- to 29.9 showed the highest prevalence of NAFLD (Fig. 3). Although more males were diagnosed with NAFLD in that group, it was not significantly different. Interestingly, a significant difference was observed between male and female children when the BMI was between 22.5–24.9 kg/m^2^.

According to Table 3, 21 (22.1%) NAFLD patients and 41 (43.2%) non-NAFLD patients were defined using both ultrasonography and FLI. Compared to the NAFLD patients diagnosed with ultrasonography but not with FLI (FLI (−) and US (+)), those defined by FLI but not with ultrasonography (FLI (+) and US (−)) were almost the same in number but were older (*P* < 0.001), had a significantly higher BMI (*P* < 0.001), higher waist circumference (*P* < 0.001) and a higher triglyceride concentration (*P* < 0.01). A significant difference was not observed in GGT, total cholesterol, LDL cholesterol, HDL cholesterol and ALT concentrations between the two groups. Further adjustment for age and sex distribution didn’t statistically change the relationships between any of these parameters.

**Table 3:** General demographic and biochemical characteristics of participants by diagnostic categories

Variables	FLI (−) and US(−)	FLI (−) and US(+)	FLI (+) and US(−)	FLI (+) and US(+)	P*	P**
Number (%)	41(43.2)	15(15.8)	18(18.9)	21(22.1)	–	
Age (yr)	10.2 ± 2.5	9.5 ± 2.5	12.1 ± 2.0	11.1 ± 2.2	*t = 3.29,p < 0.001*	
**Sex**						
Male [n (%)]	22(53.7)	10(66.7)	10(55.6)	14(66.7)	*χ*^*2*^ *= 0.42, p = 0.52*	
Female [n (%)]	19(46.3)	5(33.3)	8(44.4)	7(33.3)		
BMI (kg/m2) > 30	23.5 ± 18.5	24.6 ± 2.6	28.2 ± 3.8	27.9 ± 3.7	*t = 3.17,p < 0.001*	0.989
Waist circumference (cm) ≥94:M and ≥ 80 *cm:W*	80.3 ± 67.0	81.9 ± 6.3	93.7 ± 7.4	92.1 ± 8.1	*t = 4.87,p < 0.001*	0.642
GGT (U/L)	16.0 (14.0–21.5)	18.0(13.0–20.0)	17.5(15.8–24.0)	90.0(85.5–96.0)	z = 0.13,p = 0.89***	
Triglyceride (mg/dL)	86.0(52.0–141.5)	98.7 ± 52.9	149.5 ± 49.7	145.5 ± 62.5	*t = 2.84,p = 0.01*	0.047
Total cholesterol (mg/dL)	186.6 ± 120.0	175.6 ± 52.6	188.7 ± 41.1	214.9 ± 45.8	*t = 0.80,p = 0.43*	0.142
HDL-cholesterol (mg/dL)	50.2 ± 32.3	44.9 ± 8.7	49.4 ± 9.3	54.5 ± 10.3	*t = 1.41,p = 0.17*	0.688
LDL-cholesterol (mg/dL)	117.0 ± 40.7	111.0 ± 48.6	109.4 ± 36.6	131.3 ± 40.0	*t = 0.11,p = 0.92*	0.345
ALT (IU/L)	18.0(15.0–26.5)	34.3 ± 21.2	27.4 ± 6.8	125.5(100.0–150.8)	*t = 1.31,p = 0.20*	0.995

Values are given as means ± SD and medians (interquartile ranges) for continuous variables, and numbers (percentages) for categorical variables, *FLI* Fatty liver index, *GGT* Gamma-glutamyl-transferase, *ALT* Alanine amino transferase, *HDL* High density lipoprotein. *LDL* Low density lipoprotein

*P-For FLI (−) and US (+) versus FLI (+) and US (−), and ** P-For FLI (−) and US (+) versus FLI (+) and US (−) and were adjusted for age and sex

*** Mann-Whitney U – test was used as data were not distributed normally. *P* < 0.05 was considered significant

The multivariate analysis demonstrated that ALT above normal concentrations and higher FLI were independent risk factors correlated with the ultrasonographic NAFLD in the whole population (Table 4). Results were not compatible when the analyses were stratified by gender except for the ALT concentration in male subjects which showed a significant odds ratio. Although not specified by gender, FLI can be considered as a critical factor in the determination of ultrasonographic NAFLD.

**Table 4:** Factors associated with ultrasonographic fatty liver in different populations as determined by multivariate analysis

Variables	Odds Ratio	95% Confidence level	***P*** value
**All subjects**			
Age in years (> 11 yrs.)	0.645	0.214–.945	*p* = 0.437
Total cholesterol (> 200 mg/dL)	2.048	0.577–7.275	*p* = 0.268
HDL-cholesterol (< 40 mg/dL)	1.612	0.368–7.063	*p* = 0.526
LDL-cholesterol (> 160 mg/dL)	0.415	0.070–2.463	*p* = 0.333
ALT (> 40 IU/l)	12.785	3.054–53.514	*p* < 0.001
GGT (> 45 U/l)	1.647	0.168–16.173	*p* = 0.668
Triglycerides (> 150 mg/dl	0.443	0.123–1.592	*p* = 0.212
Fatty Liver Index	3.524	1.104–11.256	*p* = 0.033
**Male**			
Age in years (> 11 yrs.)	0.698	0.142–3.429	*p* = 0.658
Total cholesterol (> 200 mg/dL)	3.827	0.601–24.353	*p* = 0.155
HDL-cholesterol (< 40 mg/dL)	5.800	0.533–63.121	*p* = 0.149
LDL-cholesterol (> 160 mg/dL)	1.004	0.091–11.110	*p* = 0.997
ALT (> 40 IU/l)	22.071	3.476–140.147	*p* < 0.001
GGT (> 45 U/l)	2.416	0.092–63.525	*p* = 0.597
Triglycerides (> 150 mg/dl	0.159	0.019–1.364	*p* = 0.094
Fatty Liver Index	4.511	0.825–24.675	*p* = 0.082
**Female**			
Age in years	0.443	0.084–2.332	0.337
Total cholesterol (> 200 mg/dL)	0.922	0.192–4.436	0.920
HDL-cholesterol (< 40 mg/dL)	0.692	0.094–5.080	0.718
Triglycerides (> 150 mg/dl	0.907	0.165–4.973	0.911
Fatty Liver Index	4.750	0.822–27.451	0.082

*GGT* Gamma glutamyl transferase, *HDL* High density lipoprotein, *LDL* Low density lipoprotein, *ALT* Alanine amino transferase

*P* < 0.05 was considered as significant

As shown in Table 5, the discriminative ability to identify ultrasonographic NAFLD among FLI and other clinical non-invasive markers was compared using the AUROCs. The AUROC of the FLI for predicting NAFLD was 0.692. (95% CI: 0.565–0.786). Interestingly, the highest AUROC was observed for the ALT concentration with a value of 0.737 (95% CI, 0.603–0.830) followed by GGT (AUROC: 0.708, 95% CI, 0.579–0.803). When analyses were stratified by gender, the same trend was observed for males but in females, FLI had the highest AUROC. Other components of FLI showed lower AUROCs compared to FLI except for GGT.

**Table 5:** Comparison of AUROCs among non-invasive markers for predicting ultrasonographic fatty liver

Variables	AUROC	95% CI	Standard error	*P* value
**All subjects**				
FLI	0.692	0.565–0.786	0.056	< 0.001
BMI (kg/m2)	0.632	0.500–0.735	0.059	0.014
Waist circumference (cm)	0.600	0.473–0.703	0.059	0.044
GGT (U/L)	0.708	0.579–0.803	0.057	< 0.001
Triglyceride (mg/dL)	0.551	0.419–0.660	0.062	0.205
Total cholesterol (mg/dL)	0.567	0.432–0.676	0.062	0.142
HDL-cholesterol (mg/dL)	0.513	0.378–0.627	0.064	0.417
LDL-cholesterol (mg/dL)	0.556	0.426–0.634	0.061	0.179
ALT (IU/L)	0.737	0.603–0.830	0.057	< 0.001
**Male**				
FLI	0.713	0.548–0.824	0.069	< 0.001
BMI (kg/m2)	0.656	0.476–0.783	0.078	0.022
Waist circumference (cm)	0.604	0.433–0.733	0.076	0.088
GGT (U/L)	0.731	0.561–0.842	0.071	< 0.001
Triglyceride (mg/dL)	0.558	0.384–0.694	0.079	0.232
Total cholesterol (mg/dL)	0.596	0.419–0.730	0.079	0.112
HDL-cholesterol (mg/dL)	0.529	0.345–0.673	0.084	0.367
LDL-cholesterol (mg/dL)	0.591	0.418–0.722	0.077	0.121
ALT (IU/L)	0.822	0.667–0.909	0.059	< 0.000
**Female**				
FLI	0.654	0.409–0.812	0.102	0.065
BMI (kg/m2)	0.577	0.356–0.737	0.097	0.214
Waist circumference (cm)	0.573	0.336–0.741	0.104	0.242
GGT (U/L)	0.640	0.400–0.799	0.101	0.082
Triglyceride (mg/dL)	0.560	0.316–0.735	0.107	0.287
Total cholesterol (mg/dL)	0.491	0.258–0.670	0.106	0.535
HDL-cholesterol (mg/dL)	0.483	0.261–0.656	0.102	0.566
LDL-cholesterol (mg/dL)	0.483	0.257–0.659	0.103	0.565
ALT (IU/L)	0.588	0.333–0.763	0.110	0.211

*FLI* Fatty liver index, *BMI* body mass index, *GGT* Gamma glutamyl transferase, *HDL* High density lipoprotein, *LDL* Low density lipoprotein, *ALT* Alanine amino transferase

*P* < 0.05 was considered as significant

The optimal cut-off point was determined as FLI ≥ 30 with the maximum Youden index observed at 0.278 with the sensitivity and specificity, 58.33% SN, 69.49 SP, 53.85 PPV and 73.21 NPV (Table 6).

**Table 6:** Diagnostic Accuracy of the FLI

FLI Cut-Off Point	SN (%)	SP (%)	PPV (%)	NPV (%)	LR+	LR-	Youden Index
≥ 10.00	91.67	23.73	42.31	82.35	1.2019	0.3512	0.154
≥ 20.00	75.00	52.54	49.09	77.50	1.5804	0.4758	0.2754
≥ 30.00	58.33	69.49	53.85	73.21	1.912	0.5996	0.2782
≥ 40.00	44.44	79.66	57.14	70.15	2.1852	0.6974	0.2411
≥ 45.00	33.33	81.36	52.17	66.67	1.7879	0.8194	0.1469
≥ 51.00	27.78	86.44	55.56	66.23	2.0486	0.8355	0.1422
≥ 54.00	27.78	93.22	71.43	67.90	4.0972	0.7747	0.21
≥ 61.00	22.22	94.92	72.73	66.67	4.3704	0.8194	0.1714
≥ 65.00	16.67	96.61	75.00	65.52	4.9167	0.8626	0.1328
≥ 77.00	11.11	96.61	66.67	64.04	3.2778	0.9201	0.0772
≥ 81.00	5.56	96.61	50.00	62.64	1.6389	0.9776	0.0217
≥ 85.00	5.56	98.31	66.67	63.04	3.2778	0.9607	0.0386

*FLI* Fatty liver index, *SN* Sensitivity, *SP* Specificity, *LR+* Positive likelihood ratio; *LR−* Negative likelihood ratio; *PPV* Positive predictive value; *NPV* Negative predictive value

## Discussion

NAFLD is already established as one of the complications of obesity. With the current increase in the prevalence of NAFLD among children with obesity and its hepatic as well as non-hepatic consequences, the development of a simple, cost-effective and non-invasive screening method that can be used in the community in resource limited settings has become extremely important.

FLI was proven to be correlated well with NAFLD diagnosed by ultrasonography [13]) and it was also correlated with insulin resistance, coronary heart disease, and early atherosclerosis [16]. Yang et al. confirmed the significance of FLI as an indicator of ultrasonographic fatty liver and its close relationship to metabolic syndrome. They further reported its discriminative ability in identifying ultrasonographic fatty liver than other serum markers and recommended it to be used in Asians [17]. FLI was first reported by Bedogni et al. [13] based on data obtained from 216 Italian adults with and 280 without suspected liver disease. They reported accuracy of 0.84 (95% CI: 0.81–0.87). Studies published over the years provided evidence that variations in ethnicity, dietary and environmental factors, determine the cut-off values for waist circumference and BMI for Asians [18, 19] which are components in the calculations of FLI. Thus, FLI needed to be validated for different Asian ethnicities and also for the paediatric population if it is used as a screening tool in the community setting in resource limited countries in the Asian sub-continent.

The accuracy that we observed was relatively lower (AUROC = 0.692, 95% CI: 0.565–0.786) than that of Bedogni’s study but similar results were previously reported by other Asian countries such as China (AUROC = 0.721) and Korea (AUROC = 0.785) [20, 21]. However, both these studies were conducted in adult populations. Another study that recruited over 8000 Chinese adults reported an AUROC of 0.834 which was similar to what was reported by Bedogni et al. and the AUROC for FLI was also significantly greater than that of individual components used in the calculation of FLI. Furthermore, higher sensitivity and specificity was associated with the optimum cut-off point calculated for that population compared to the studies reported from Asia. It may be attributed to the higher sample size used in the study sample. Another reason would be the different risk factors associated with different Asian populations. It was also interesting to note that the accuracy of FLI in the diagnosis of NAFLD was higher and satisfactory in most Caucasian populations where AUROC was reported to be between 0.813–0.930 [22].

Although limited data are available on the validity of FLI in the paediatric population, a study conducted in the Netherlands on obese children revealed that it had moderate diagnostic accuracy (AUROC = 0.71, 95% CI: 0.61–0.80) in severely obese children and adolescents in the diagnosis and exclusion of NAFLD [23]. Although children recruited to our study were not severely obese, these results are comparable to the results obtained in our study. Another reason for the lower values observed for sensitivity, specificity and Youden index would be the comparatively low sample size and analogous age of children recruited to the study despite having higher triglyceride, GGT levels and a higher prevalence of NAFLD. However, the objective of our study was to determine whether it can be used as a screening tool in the community setting, especially during school health inspections which are conducted annually, to screen children for NAFLD and to follow them up further if they belong to the high risk category and not as a diagnostic tool. The optimal cut-off point of the FLI identified for the screening of NAFLD which was defined by the maximum Youden index was 30. Our results are comparable to results reported among Chinese study participants and in the original study by Bedogni et al. where they also obtained 30 as the cut-off value [12, 13].

Non-invasive prediction scores are of importance in resource limited settings for clinicians to screen NAFLD rapidly and easily. Accurate exclusion of the presence of NAFLD in high risk groups such as obese children would allow clinicians to minimize the occurrence of metabolic syndrome and other complications of NAFLD during their adolescence and adulthood. Since widely accepted criteria to be met for a clinically useful test to exclude a disorder include a sensitivity > 95% and a negative likelihood ratio of < 0.10, the results that we obtained do not meet this degree of diagnostic accuracy with a sensitivity of 58.33% and 0.59. It was interesting to note that the prediction scores published by Bedogni et al. also didn’t meet this degree of accuracy where sensitivity varied between 82 and 85% and negative likelihood ratio between 0.17–0.25 [13]. The smaller sample size used in our study population would have contributed to the low sensitivity and the higher negative likelihood ratio detected.

Our study also revealed that overweight or obese children diagnosed with NAFLD ultrasonographically or screened by FLI had worse metabolic status comparatively. Compared to children who were not diagnosed with NAFLD, BMI, GGT, and ALT concentrations were significantly different in the children who were diagnosed with NAFLD ultrasonographically. Besides, FLI identified waist circumference and triglyceride concentration to be significantly different between children with and without NAFLD. FLI was calculated based on metabolism-related parameters such as triglyceride concentration, waist circumference, BMI and GGT. Therefore, extra attention must be drawn to metabolic control of the above parameters and the management of NAFLD to prevent any irreversible complications related to NAFLD.

We believe that this is the first study carried out in South Asian obese children to validate the accuracy of FLI to screen NAFLD in the community setting. However, our study is associated with some limitations. The sample size of the study is comparatively smaller and ultrasonography which is widely used in Sri Lanka to diagnose NAFLD was used as the reference standard although it has some limitations. However, according to previous reports, ultrasonography was recognized as the most practical and feasible option to diagnose NAFLD especially in resource limited settings, clinical practice and epidemiological studies [24].

In conclusion, the accuracy of FLI was validated against ultrasonography in overweight and obese children aged 5–15 years and the optimum cut-off value of the FLI to screen for NAFLD was determined at 30. The accuracy of the FLI was comparable to that reported from other Asian countries and was lower than Caucasian populations. Although the applicability of FLI in the clinical diagnosis is limited, it can be used as a screening tool in the community setting and in epidemiological studies in Sri Lanka to identify obese children who are at a higher risk; therefore, to prevent any metabolic, cardiovascular and renal complications but most importantly to prevent the progression of NAFLD.

## Data Availability

The datasets used and/or analyzed during the current study are available from the corresponding author on reasonable request.

## Abbreviations

FLI: Fatty Liver Index
NAFLD: Non-alcoholic fatty liver disease
AUROC: Receiver operator characteristic curve
ALT: Alanine aminotransferases
HDL-C: High-density lipoprotein cholesterol
BMI: Body Mass index
CDC: Centers for Disease Control and Prevention
PNPLA3: Patatin-like phospholipase domain containing protein 3
